# Rapid Prediction of Nonlinear Effective Properties of Complex Microstructure Lattice Materials

**DOI:** 10.3390/ma18061301

**Published:** 2025-03-15

**Authors:** Jun Yan, Zhihui Liu, Hongyuan Liu, Chenguang Zhang, Yinghao Nie

**Affiliations:** 1 State Key Laboratory of Structural Analysis, Optimization and CAE Software for Industrial Equipment, Department of Engineering Mechanics, International Research Center for Computational Mechanics, Dalian University of Technology, Dalian 116024, China; yanjun@dlut.edu.cn (J.Y.); liuzhihui@mail.dlut.edu.cn (Z.L.); ayangx@mail.dlut.edu.cn (H.L.); zhangcg@mail.dlut.edu.cn (C.Z.); 2 Ningbo Institute of Dalian University of Technology, Ningbo 315033, China

**Keywords:** lattice materials, FEM-Cluster-based analysis, reduced-order model, nonlinear effective properties

## Abstract

Lattice materials are renowned for their exceptional mechanical performance and transformative potential for aerospace and structural engineering applications. However, current research primarily focuses on the effective elastic properties of lattice microstructures, whereas there are few studies on the prediction of their effective nonlinear properties, thus limiting the practical application of lattice materials. In addition, the characterization of complex micro structured lattice materials often requires the generation of many elements and performing nonlinear finite element analysis, which involves high computational costs. To address these challenges and enable the efficient prediction of the nonlinear effective properties of complex lattice microstructures in heterogeneous materials, the FEM-Cluster-based Analysis (FCA) approach is proposed. In the offline phase, a reduced-order model and offline database are established. In the online phase, the principle of the cluster minimum complementary energy incremental algorithm is used to rapidly predict the nonlinear effective properties of heterogeneous materials. This method is applied to conduct extensive comparisons with direct numerical simulation across two-dimensional and three-dimensional lattice materials to demonstrate that FCA can achieve similar accuracy while significantly enhancing computational efficiency, thereby offering promising potential for optimizing lattice material design in structural applications.

## 1. Introduction

In modern engineering fields such as aerospace, structural design typically focuses on weight reduction while maintaining performance, with an overall trend toward integrating materials, structures, and functionality [1]. Lattice materials typically refer to microstructures with periodic trusses [2] and have attracted significant attention because of their excellent mechanical and physical properties, including lightness, high stiffness- and strength-to-weight ratios, vibration damping, energy absorption, and high porosity [3]. These material properties make them particularly promising for different applications.

The research on lattice materials has expanded in several ways. For example, Ashby and Bréchet [4] compared lattice materials with foam materials and found that they significantly outperformed foams in terms of their modulus characteristics. Yin et al. [5] highlighted the multifunctional design potential of lattice sandwich structures in fundamental research and practical applications. Yan et al. [6] found that ultralight X-shaped lattice sandwich panels exhibited good thermal and mechanical bearing capacities. Furthermore, researchers have discovered that lattice materials show promise for applications such as electromagnetic wave absorption [7], soundproofing [8], and zero/negative thermal expansion [9]. These new structural materials, which combine lightweight properties with multifunctionality, provide novel solutions for the integrated design of high-performance components [10].

To improve the application of lattice materials and reduce the experimental costs, several methods have been developed to address the equivalent performance issues of this microstructure. In terms of theoretical analysis, methods such as Eshelby’s theory [11], self-consistent and generalized self-consistent methods [12,13], and the Hashin–Strikman variational approach [14,15] can be used to evaluate heterogeneous materials and provide upper and lower bounds for their effective elastic properties. With the rapid advances in computer technology, several numerical methods have been developed to determine the effective properties of lattice materials. These include approaches such as the Representative Volume Element (RVE) method [16,17], asymptotic homogenization methods [18], and the Novel Implementation of Asymptotic Homogenization (NIAH) [19,20]. Building on the RVE method, Pecullan et al. [21] investigated the influence of the Dirichlet and Neumann boundary conditions on the effective properties of RVEs. Yan et al. [22] explored the underlying mechanisms of these effects and revealed that the deformation and stress responses at the boundaries under Dirichlet or Neumann conditions differed from those in the actual microstructure of the lattice. The advantage of homogenization-based numerical methods is their efficiency in predicting the effective properties of heterogeneous materials, albeit at the expense of accuracy. However, these methods typically involve certain assumptions or limitations and are not capable of predicting the local nonlinear effective properties of complex microstructures.

For the nonlinear effective properties of heterogeneous materials, direct numerical simulation methods such as the finite element method (FEM) [23] and Fast Fourier Transform (FFT) [24,25] are commonly used. For complex-shaped lattice materials, it is generally necessary to divide many elements, and finite element calculations require a considerable amount of time [26]. This problem is particularly evident when nonlinear structural analyses are conducted. FFT methods typically require the introduction of homogeneous reference materials, and the parameters selected may affect the accuracy and convergence of the entire algorithm. They are highly dependent on the reference materials selected, and convergence problems can occur, particularly when considering materials with high-porosity [27] such as lattice materials. Therefore, to advance their mechanical analysis, enhancing the computational efficiency of complex lattice materials is a problem that needs to be addressed.

A range of model reduction techniques has been developed and applied to address the long computation times associated with traditional numerical methods. These include Proper Orthogonal Decomposition (POD) [28,29], Proper Generalized Decomposition (PGD) [30], Transformation Field Analysis (TFA) [31], and Nonuniform TFA (NTFA) [32]. Another approach involves data-driven clustering-based model-reduction techniques. The fundamental principle of these methods is to divide the RVEs into clusters based on the similarity of their material properties. Notable examples of these reduced-order modeling techniques include Self-consistent Clustering Analysis (SCA) [33,34] and Virtual Clustering Analysis (VCA) [35,36]. In addition, some methods combine neural networks, such as Deep Neural Networks (DNNs) [37] and Long Short-term Memory (LSTM) [38], to analyze the nonlinear equivalent properties of microstructures.

Recently, Cheng et al. [39] proposed a method called the FEM-Cluster-based Analysis (FCA), which efficiently and accurately predicts the nonlinear effective properties of an RVE through a reduced-order model. The FCA method comprises offline and online phases. In the offline phase, the RVE is divided into several clusters using the k-means clustering algorithm based on the strain concentration tensor. A reduced-order model is constructed based on the interaction matrix. An interaction matrix relating the cluster eigenstrain and cluster stress is established by solving the finite element equations of the RVE in the elastic range. In the online phase, the reduced-order model and incremental algorithm of the Principle of Cluster Minimum Complementary Energy (PCMCE) is used to predict the nonlinear properties of heterogeneous materials [40]. Furthermore, Nie et al. [41] introduced an innovative method for reconstructing the D matrix using singular value decomposition, which reduces the nonlinear prediction errors for heterogeneous materials. This algorithm is also applicable for the rapid nonlinear effective property analysis of porous materials.

Current studies on the rapid elastoplastic analysis of lattice materials is limited [26]. Owing to the high porosity inherent in lattice materials, reduced-order methods based on reference materials in micromechanics cannot be readily extended to nonlinear analyses, which may encounter difficulties in convergence [27]. Although the FCA constructs reduced-order models based on the RVE of the actual material for elastoplastic analysis thereby avoiding these problems, the complex microstructures and wide range of porosity variations in lattice materials pose significant challenges. The applicability of FCA for predicting the effective elastoplastic properties of such complex lattice materials requires investigation, and there are few studies that compare computational efficiency and accuracy. Therefore, in this study, FCA is introduced for the elastoplastic analysis of complex lattice materials and a series of numerical studies and comparisons are conducted. The mechanical behaviors of different two-dimensional (2D) and three-dimensional (3D) lattice materials are compared with intricate microstructures. This includes analyzing the effective stiffness matrices in the linear elastic stage and the distribution of the effective stress–strain curves in the nonlinear stage. The mechanical performance distribution patterns of different microstructures are explored, providing valuable insights and references for practical applications.

The remainder of this paper is organized as follows. Section 2 introduces the concept of nonlinear homogenization and outlines the solution framework for implementing the FCA. In Section 3, the modeling of complex micro structured lattice materials is presented. Section 4 focuses on the rapid and effective analysis of the 2D and 3D lattice materials and investigates the stress–strain response of the RVE lattice material. The accuracy and efficiency of the method are validated using direct numerical simulation (DNS), and the results are discussed. Finally, Section 5 summarizes the findings and outlines the directions for future research.

## 2. Methodology and Solution Framework

The Hill–Mandel condition [12,42] is widely used for the analysis of composites [43] and porous materials [44]. In this section, the Hill–Mandel condition for the analysis of lattice materials is reviewed. The fundamental theory of the FCA method is then introduced, and a computational process is presented for predicting the nonlinear effective properties of lattice materials. Given that lattice materials typically contain many pores, the following section starts with an introduction to the fundamentals of the homogenization of lattice materials.

### 2.1. Homogenization of Lattice Materials

According to homogenization theory, the macroscopic properties of periodic heterogeneous materials can be predicted and characterized using information from the microstructural RVE. The microstructural RVE can be equivalently represented on the macroscopic scale as a homogeneous material with the same area. This effective homogeneous material must satisfy the Hill–Mandel condition, which states that the average strain energy density over the RVE is equal to the product of the average stress and strain. This condition can be expressed mathematically as

(1) $$\langle \sigma :\varepsilon \rangle_{\Omega }=\langle \sigma \rangle_{\Omega }:\langle \varepsilon \rangle_{\Omega }$$

(2) $$\langle *\rangle_{\Omega }=\frac{1}{\left|\Omega \right|}\int_{\Omega }(*)d\Omega$$

where ***σ*** and ***ε*** are the stress and strain, respectively, and $\langle *\rangle_{\Omega }$ is the definition of the averaging symbol.

**Figure 1:**
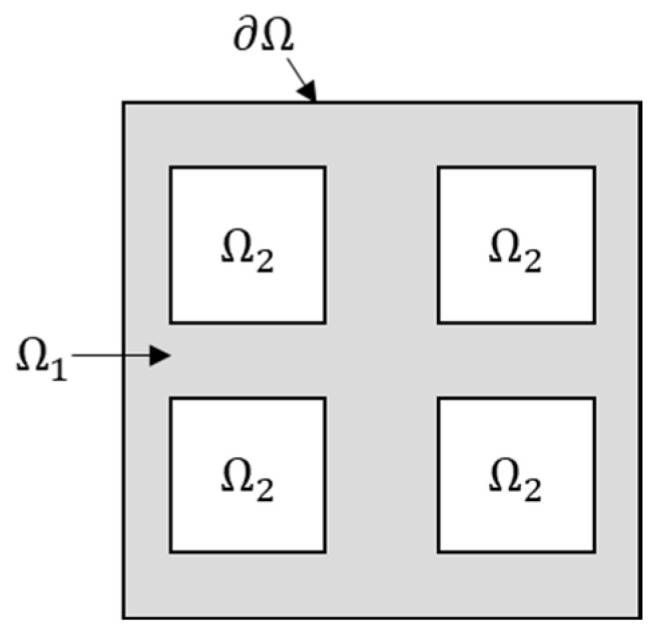
Example of lattice material RVE.

Based on a previous study [41], for a lattice material RVE, as shown in Figure 1, with an RVE under uniform displacement boundary conditions, the average strain is

(3) $$\begin{array}{ll} \langle \varepsilon \rangle_{\Omega } & =\frac{1}{2|\Omega |}\int_{\Omega }\left(\nabla u+(\nabla u{)}^{T}\right)d\Omega \\ & =\frac{1}{2|\Omega |}\left\{\int_{\Omega_{1}}\left(\nabla u+(\nabla u{)}^{T}\right)d\Omega_{1}+\int_{\Omega_{2}}\left(\nabla u+(\nabla u{)}^{T}\right)d\Omega_{2}\right\}\\ & =\frac{1}{2|\Omega |}\left\{\int_{\partial \Omega }\left(\left({\bar{\varepsilon }}^{0}\cdot x\right)⊗n+n⊗\left({\bar{\varepsilon }}^{0}\cdot x\right)\right)d\partial \Omega +\int_{\partial \Omega_{1}\cap \partial \Omega_{2}}(u⊗n+n⊗u)d\partial \Omega \right\}\\ & ={\bar{\varepsilon }}^{0}+\frac{1}{2|\Omega |}\left\{\int_{\partial \Omega_{1}\cap \partial \Omega_{2}}(u⊗n+n⊗u)d\partial \Omega \right\} \end{array}$$

where $\Omega_{1}$ is the matrix domain; $\Omega_{2}$ is the pore domain; the entire domain is represented by $\Omega =\Omega_{1}\cup \Omega_{2}$; u is the displacement; ${\bar{\varepsilon }}^{0}$ is the given constant strain; and ∂Ω, $\partial \Omega_{1}$ and $\partial \Omega_{2}$ are the corresponding boundary differential parts.

Owing to the presence of pore strain, for lattice materials, the average strain of the RVE can be expressed as

(4) $$\langle {\varepsilon \rangle }_{\Omega }=\frac{1}{\left|\Omega \right|}\int_{\Omega_{1}}\varepsilon {d\Omega }_{1}+\frac{1}{\;\left|\Omega \right|}\int_{\partial \Omega_{2}}\left(u⨂n\right)d\partial \Omega_{2}$$

In Equation (4), the first term can be computed using finite element analysis (FEA) or the cluster-averaged reduced-order model method. However, the second term requires the displacement at the pore boundary nodes to be calculated, which is only feasible using full-field DNS solutions. For reduced-order model methods, in which cluster stresses and strains are the basic variables, the second term is challenging to compute because the displacements at the pore boundary are difficult to determine.

However, a previous study [41] reports that the energy equivalence principle can be used for effective modeling. In the plastic stage, the incremental energy equivalence relationship is used to calculate the average stress at each incremental step for the lattice materials. Based on this derivation, the effective stress of the lattice material RVE at step n+1 can be expressed as

(5) $$\sigma_{ij}^{n+1}=\frac{2\left\{\frac{\Delta \Phi^{n}}{\left|\Omega \right|}-{\left(\sigma^{n}\right)}^{T}\Delta \varepsilon^{n}\right\}}{\;\Delta \varepsilon_{ij}^{n}}+\sigma_{ij}^{n}$$

where the relationship between the increment of porous strain energy and effective stress is represented by $\Delta \Phi^{n}=\frac{1}{2}{\left(\Delta \sigma^{n}\right)}^{T}\left|\Omega \right|\Delta \varepsilon^{n}+{\left(\sigma^{n}\right)}^{T}\left|\Omega \right|\Delta \varepsilon^{n}$. This equation allows for the calculation of both the linear and nonlinear effective properties of the RVE.

### 2.2. FCA Method for Solving the Nonlinear Effective Properties of Lattice RVEs

The fundamental governing equations of the FCA method for predicting the nonlinear effective properties of lattice materials are first introduced. The FCA method is divided into offline and online phases. In the offline phase, a reduced-order model is constructed based on elastic FEA and a clustering algorithm to construct an offline database. In the online phase, a reduced-order model is used combined with an incremental algorithm based on the PCMCE incremental algorithm to predict effective nonlinear properties [39,40,41].

#### 2.2.1. The Governing Equations of FCA

The FCA governing equations for the 2D RVE defined over the domain $\Omega =\{\left(x_{1},x_{2}\right)|0\leq x_{1}\leq 1,0\leq x_{2}\leq 1\}$ are

(6) ∇⋅σ(x)=0,     x∈Ω

(7) $$\varepsilon =\nabla^{\;sym}u(x),\;\;\;\;\;x\in \Omega$$

(8) $$\sigma \left(x\right)=\{\begin{array}{ll} C\left(x\right):\varepsilon^{\prime } & \;\mathrm{Elastic}\;\mathrm{Range}\\ f\left(x,\varepsilon^{\prime },\omega \right) & \;\mathrm{Nonlinear}\;\mathrm{Range} \end{array}$$

(9) $$\varepsilon^{\prime }=\varepsilon -\varepsilon^{*}$$

(10) $${u_{i}|}_{x=0}={u_{i}|}_{x=1},{t_{i}|}_{x=0}={-t_{i}|}_{x=1},\;\;\;\;\;\;\;\;\;\;i=1,2$$

(11) $${u_{i}|}_{\left(x_{1}=0.5,x_{2}=0.5\right)}=0$$

where Equations (6)–(8) represent the equilibrium, strain–displacement geometry, and constitutive equations, respectively; C(x) is the elastic stiffness matrix; $f\left(x,\varepsilon^{\prime },\omega \right)$ is a nonlinear function whose parameters correspond to different plastic strengthening criteria; and in Equation (9), $\varepsilon^{\prime },\varepsilon$, and $\varepsilon^{*}$ are the mechanical strain, total strain, and eigenstrain, respectively. Equation (10) indicates that the RVE model applies periodic boundary conditions. The rigid-body displacement was constrained by fixing the degrees of freedom at a single point, as shown in Equation (11). The boundary value problem for the RVE can be solved by applying the eigenstrain $\varepsilon^{*}$.

#### 2.2.2. Offline Phase

First, the elastic FEA for the RVE is performed to compute the strain concentration tensor **A**. Under periodic boundary conditions, a uniform eigenstrain is applied across the entire domain of the RVE, and the strain responses in each direction for all elements are calculated. The finite element calculation is

(12) $$KU^{mn}=F^{mn}$$

where K is the total stiffness matrix of the RVE; $U^{mn}$ is the nodes displacement vector; and $F^{mn}$ is the “external force” vector applied at the nodes of the RVE model, which is derived from the uniformly applied eigenstrain.

This allows for the establishment of the relationship between the microscopic elastic strain $\varepsilon^{\mathrm{micro}}\left(x\right)$ and the homogeneously elastic macroscopic strain $\varepsilon^{\mathrm{macro}}\left(X\right)$.

(13) $$\varepsilon^{\mathrm{meso}}\left(x\right)=A\left(x\right):\varepsilon^{\mathrm{macro}}\left(X\right),\;x\in \Omega_{\mathrm{meso}}$$

Next, the similarity between two material points and their corresponding elements is assessed using the Frobenius norm in conjunction with the strain concentration tensor. The equation is

(14) $$\|A\left(x\right)-A\left(x\right){\|}_{F}=\sqrt{\underset{i=1}{\sum }\underset{j=1}{\sum }{\left(A_{ij}\left(x_{1}\right)-A_{ij}\left(x_{2}\right)\right)}^{2}}$$

where in the 2D problem, i,j=1, 2 or 3, and in the 3D problem, i,j=1, 2, 3, 4, 5 or 6.

A standard K-means clustering algorithm is used to reduce the complexity of the model by dividing it into clusters. The elements in the RVE model with similar mechanical responses (strain concentration tensors) and identical material properties in the elastic phase are grouped using a clustering algorithm. The similarity between the elements is then defined based on the norm of the strain concentration tensor. Final clustering is achieved by minimizing the total distance Y from the strain concentration tensor of each element to the corresponding cluster center.

(15) $$Y=\underset{Y^{\prime }}{\arg\;\min}\overset{n_{c}}{\underset{I=1}{\sum }}\underset{e\in \Omega^{I}}{\sum }\|A_{e}-{\bar{A}}^{I}{\|}^{2}$$

where $A_{e}$ is the strain concentration tensor for the e-th element, ${\bar{A}}^{I}$ is the average strain concentration tensor for all elements in the I-th cluster, and $\Omega^{I}$ and $n_{c}$ are the total number of clusters.

Finally, using the previously constructed cluster model, the cluster stress and cluster strain are defined as the basic variables to solve the interaction matrix. The FEM is used to compute the interaction matrix for the RVE under uniform cluster eigenstrain loads. By applying a unit eigenstrain to each cluster of the cluster model, the average stress of the remaining clusters is calculated, forming submatrices of the interaction matrix. For the 3D structure, this process is performed in six directions corresponding to six loading conditions. This procedure is repeated for each cluster, ultimately constructing a complete interaction matrix, denoted as D, and its components are expressed as

(16) $$D_{ijkl}^{IJ}=\frac{1}{\Omega^{I}}\underset{e\in \Omega^{I}}{\sum }\sigma_{ij,e}^{\left(\Psi \right)}\;{\hat{\varepsilon }}_{\Lambda ,kl}^{*\left(\Psi \right),J}\left|\Omega_{e}\right|$$

where in the 2D problem, Ψ=1, 2, or 3, and ij=11, 22, or 12, and in the 3D problem, Ψ=1, 2, 3, 4, 5, or 6, and ij=11, 22, 33, 23, 13, or 12; $D_{ijkl}^{IJ}$ is interaction matrix components; $\sigma_{ij,e}^{\left(\Psi \right)}$ is the stress vectors of each cluster in the cluster model; ${\hat{\varepsilon }}_{\Lambda ,kl}^{*\left(\Psi \right),J}$ is the eigenstrain of the unit cluster under the applied working condition Ψ.

The interaction matrix must be reconstructed to consider the complex microstructure of lattice materials and avoid the influence of small singular values on the interaction matrix properties [41]. This involves singular value decomposition on the original interaction matrix.

(17) $$D=Q\Lambda^{D}P^{T}$$

where the singular value matrix is denoted as $\Lambda^{D}=\mathrm{diag}\left(\lambda_{1}^{D},\ldots ,\lambda_{k}^{D},\ldots ,\lambda_{m}^{D}\right),\;\lambda_{1}^{D}\geq \lambda_{2}^{D}\geq \ldots \geq \lambda_{m}^{D}$, while Q and P are the orthogonal matrices of the singular vectors of matrix D. By conducting an energy norm error analysis on the effective properties of the heterogeneous material, the singular value matrix $\Lambda^{*}=\mathrm{diag}\left(\lambda_{1}^{D},\ldots ,\lambda_{k}^{D},0,\ldots ,0\right),\;\lambda_{1}^{D}\geq \lambda_{2}^{D}\geq \ldots \geq \lambda_{k}^{D}$, is obtained which retains a rank of k.

This allows for the reconstruction of the interaction matrix:

(18) $$D^{*}=Q\Lambda^{*}P^{T}$$

where D* is the reconstructed interaction matrix.

The interaction matrix serves as the foundation for subsequent calculations of the nonlinear effective properties. The final effective analysis model requires the input of the structure’s linear elastic matrix C. This can be achieved using the properties of the interaction matrix, which are calculated as

(19) $$C=\frac{1}{\left|\Omega \right|}\overset{n_{c}}{\underset{I=1}{\sum }}\overset{n_{c}}{\underset{J=1}{\sum }}D^{IJ}\left|\Omega^{I}\right|$$

For symmetric structures, the components of the effective elastic matrix for the 2D plane stress are given by

(20) C=[C11C120 C220sym C44]

The components of the effective elastic matrix for the 2D plane strain are given by

(21) C=[C11C12C130 C22C230  C330sym  C44]

The components of the effective elastic matrix for 3D analysis are given by

(22) C=[C11C12C13000 C22C23000  C33000   C4400    C550sym    C66]

#### 2.2.3. Online Phase

In the online phase, this is achieved using an online incremental algorithm based on the PCMCE [40]. The principle is extended to a cluster form by introducing an approximation where the cluster complementary energy density is used to replace the integral of the complementary energy density at each point. The PCMCE states that among all statically permissible cluster stress vectors, the true cluster stress vector minimizes the total complementary energy of the reduced-order model. Due to the properties of the interaction matrix, for any given column vector α, a parametric static admissible cluster stress can be obtained. By substituting this vector into the cluster minimum complementary energy, the PCMCE for the reduced-order FCA model can be derived as

(23) $$\alpha_{\mathrm{opt}}=\underset{\alpha }{\min}\Pi_{c}\left(\alpha ,\Delta {\hat{\varepsilon }}^{*}\right)=\underset{\alpha }{\min}\left\{\frac{1}{2}\alpha^{T}D^{T}VS^{\prime }D\alpha +\alpha^{T}D^{T}V\Delta {\hat{\varepsilon }}^{*}\right\}$$

where $S^{\prime }$ is the elastoplastic compliance matrix, V is the volume diagonal matrix of each cluster, and $\Delta {\hat{\varepsilon }}^{*}$ is the incremental eigenstrain.

Next, by combining the reconstructed interaction matrix, the PCMCE incremental algorithm is updated and the optimization problem of the PCMCE under incremental loading is solved.

(24) $$\alpha_{\mathrm{opt}}^{*}=\underset{\alpha }{\min}\Pi_{c}^{*}\left(\alpha ,\Delta {\hat{\varepsilon }}^{*}\right)=\underset{\alpha }{\min}\left\{\frac{1}{2}\alpha^{T}D^{*T}VS^{\prime }D^{*}\alpha +\alpha^{T}D^{*T}V\Delta {\hat{\varepsilon }}^{*}\right\}$$

In each incremental step, the algorithm seeks a cluster stress increment that approximates the equilibrium and a cluster mechanical strain that ensures compatibility, ultimately enabling the determination of the nonlinear effective properties $\langle {\hat{\sigma }}_{k+1}\rangle_{\Omega }$ of the microstructure.

(25) $$\Delta {\hat{\sigma }}_{k}=D\alpha_{\mathrm{opt}}^{*}$$

(26) $$\langle {\hat{\sigma }}_{k+1}\rangle_{\Omega }=\langle {\hat{\sigma }}_{k}+\Delta {\hat{\sigma }}_{k}\rangle_{\Omega }=\frac{1}{\left|\Omega \right|}\underset{e\in \Omega^{I}}{\sum }{\hat{\sigma }}_{k+1}\left|\Omega^{I}\right|$$

where $\Delta {\hat{\sigma }}_{k}$ is the stress increment of step k, ${\hat{\sigma }}_{k}$ is the stress of step k, and ${\hat{\sigma }}_{k+1}$ is the stress increment of step k+1.

### 2.3. Solution Framework

For lattice materials, the steps based on the nonlinear effective properties of the FCA are as follows:

Step 1: use commercial software to construct a geometric model of lattice materials, and then perform mesh division to obtain the FE model of RVE;

Step 2: apply periodic boundary conditions to the lattice RVE and load eigenstrain to calculate the strain concentration tensor A;

Step 3: clustering the RVE model based on the FCA clustering algorithm and constructing a lattice RVE cluster model;

Step 4: apply eigenstrain to each cluster to construct an interaction matrix $D^{IJ}$ and calculate the volume fraction $v^{I}$ of each cluster; meanwhile, construct a reconstruction interaction matrix based on the reconstruction algorithm, form an offline database, and construct an offline reduced-order model;

Step 5: use the online incremental algorithm PCMCE and combine it with an offline database to solve the cluster minimum complementary energy problem and obtain the column vector $\alpha_{\mathrm{opt}}$;

Step 6: use Equation (25) to calculate the stress increment, update the nonlinear effective properties of the next step lattice material until the increment step is completed, and obtain the final nonlinear effective properties of the lattice material.

**Figure 2:**
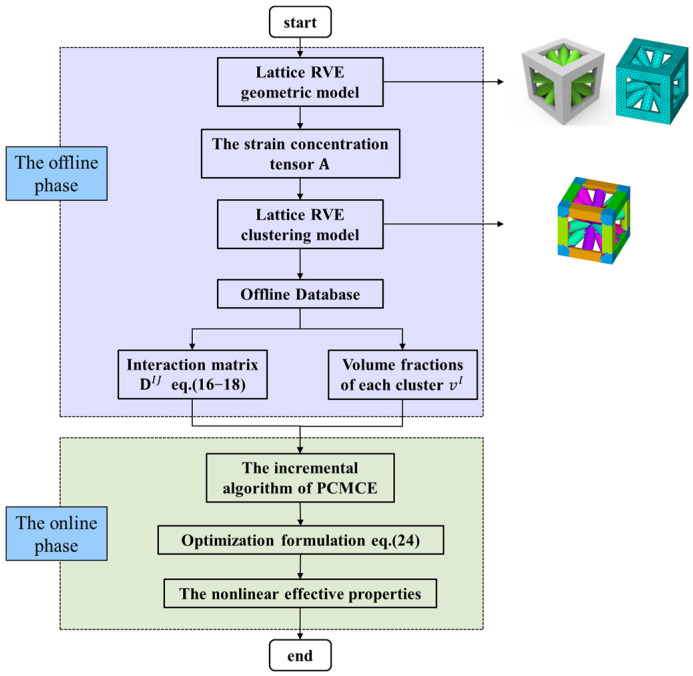
FCA calculation framework for lattice materials.

The specific flowchart of this process is shown in Figure 2.

In this study, the commercial software ANSYS 2024 R1 was used for offline FEA; and MATLAB R2024a software was used for offline clustering and online effective property calculations.

## 3. Modeling of Complex Micro Structured Lattice Materials

The modeling of complex micro structured lattice materials is conducted in both 2D and 3D configurations. The material is modeled using an elastoplastic constitutive relationship. The modeling approach and methodology are described below.

### 3.1. Introduction to 2D Model of Lattice Material

**Figure 3:**
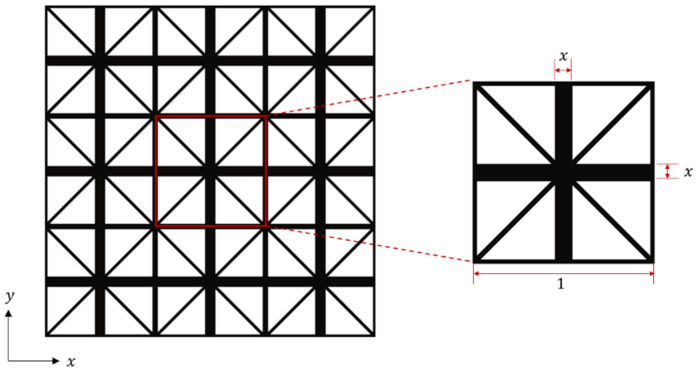
2D lattice material and its microstructural RVEs.

This study uses the 2D lattice material proposed by Wang and McDowell [45], which exhibits excellent in-plane mechanical properties. As shown in Figure 3, the microstructure of this lattice material consists of a square frame connected by four rod elements.

**Figure 4:**
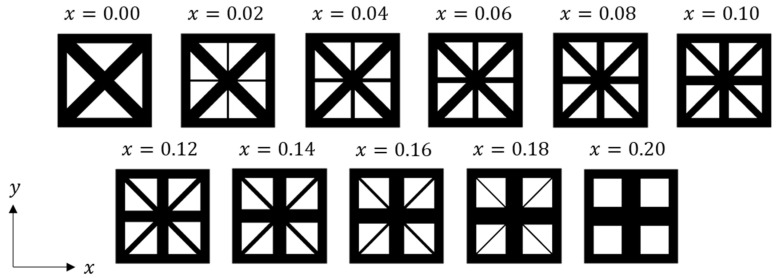
2D lattice microstructural RVEs with varying parameter x.

A lattice material model is constructed based on the parameterization approach proposed by Wang [46]. The microstructural RVE is set to a size of 1 × 1. By controlling the material volume in the RVE, the material volume fraction is maintained at 64% within the square frame. The width parameters of the horizontal and vertical rods in the center, denoted as *x*, are varied within the range of 0≤x≤0.2. A series of 2D lattice RVEs was then constructed, as shown in Figure 4.

### 3.2. Introduction to 3D Model of Lattice Material

**Figure 5:**
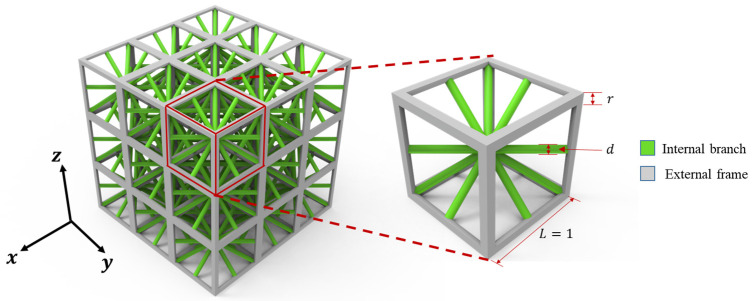
Schematic diagram of GBCC-enhanced configuration.

Based on the modeling approaches found in a previous study [47], the graded body-centered cubic (GBCC) lattice material model [48] was enhanced in this study and consists of a microstructural RVE with 8 central cylindrical rods and 12 fan-shaped cylindrical rods. Additionally, diagonal rods were introduced to connect the points on the frame, resulting in a total of twenty rods in the central part of the RVE, forming a GBCC-enhanced configuration. The geometric parameters of the microstructure include three variables: the frame edge length L=1, radius r of the fan-shaped cylindrical rods, and diameter d of the central cylindrical rods. Its structure is illustrated in Figure 5.

The relative density β of the microstructure is defined as the ratio of the lattice material density to the solid material density. The specific formula is as follows:

(27) $$\beta =\frac{V_{strut}}{V_{lattice}}$$

where $V_{strut}$ is the volume of the internal struts that form the lattice microstructure, and $V_{lattice}$ is the envelope volume enclosed by the external boundary of the lattice microstructure. By varying the radius r of the framework and diameter d of the struts, a series of lattice microstructures with different relative densities can be obtained.

Additionally, by altering the r/d ratio while keeping the relative density constant, lattice microstructures with different aspect ratios $\zeta_{exter}$ can be obtained, where

(28) $$\zeta_{exter}=\frac{V_{exter}}{V_{strut}}$$

and $V_{exter}$ is the volume of the external frame.

**Figure 6:**
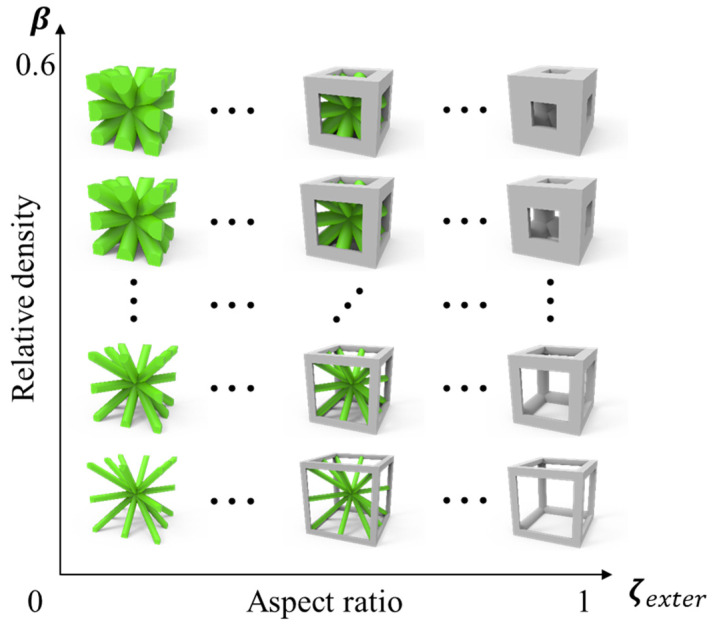
Microstructure RVEs with different aspect ratios and relative densities.

By combining these two descriptions, a set of 3D lattice material models can be generated. Microstructural lattice RVEs with different relative densities and topological configurations are fabricated by systematically varying the control parameters. Figure 6 illustrates this, with the horizontal axis representing $\zeta_{exter}$ and the vertical axis representing β, which correspond to different microstructural geometries.

### 3.3. Introduction to Complex Voxel Lattice Modeling

Based on previous studies [49,50], it is evident that voxel grid (VG) modeling significantly improves the efficiency of solving the interaction matrix in the offline phase. In addition, VGs play a crucial role in setting material density during structural optimization. This approach lays the foundation for the subsequent optimization of lattice materials.

**Figure 7:**
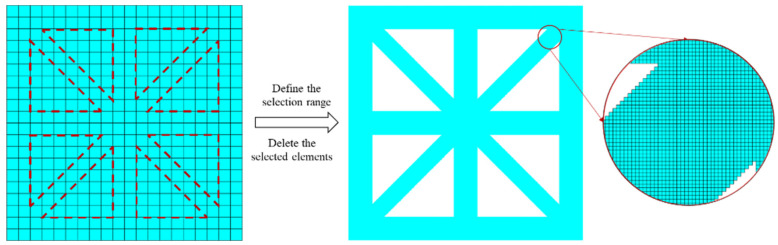
Voxel grid (VG) modeling process for lattice materials.

In this study, a 2D complex lattice with a parameter x=0.10 is selected. Initially, a regular grid with 500 × 500 elements is constructed. The coordinates of the corner points of the configuration are then determined. Next, elements within the region defined by the corner points are selectively removed. The remaining elements form the lattice material, which comprises 160,600 elements with a volume fraction of 0.6424. Figure 7 illustrates this process.

### 3.4. Material Constitutive Parameters

In this study, a multilinear elastoplastic constitutive model is used. The material properties of the linear elastic phase are characterized by an elastic modulus of 200 MPa and Poisson’s ratio of 0.3.

(29) E=200 MPa, ν=0.3

The yield stress is 0.4 MPa, and the plasticity is governed by the von Mises isotropic hardening criterion

(30) $$f=\hat{\sigma }-\sigma_{Y}\left(\hat{\varepsilon }\right)\leq 0$$

where $\hat{\sigma }$ is the von Mises effective stress, while the yield stress $\sigma_{Y}$ is determined using the hardening law, which depends on the effective plastic strain $\hat{\varepsilon }$.

The hardening law is piecewise linear and isotropic:

(31) $$\sigma_{Y}\left(\hat{\varepsilon }\right)=\{\begin{array}{cc} 0.4+10\hat{\varepsilon } & \hat{\varepsilon }\in \left[0,0.01\right)\\ 0.5+2\hat{\varepsilon } & \hat{\varepsilon }\in \left[0.01,\infty \right) \end{array}\mathrm{MPa}$$

## 4. Comparison and Discussion of Elastoplastic Effective Properties

In this section, based on the models established in the previous section, an offline analysis is conducted for 2D, 3D, and voxelized lattice material models. The interaction matrix properties are used to obtain an effective elastic stiffness matrix. The anisotropic distributions across different lattice configurations are compared, and further analysis of the nonlinear effective properties of the complex micro structured lattice materials is performed using the FCA method.

### 4.1. 2D Results of Effective Properties Analysis

**Figure 8:**
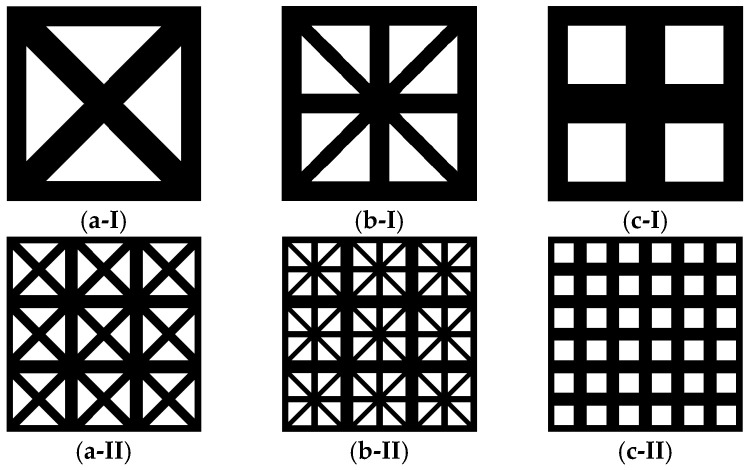

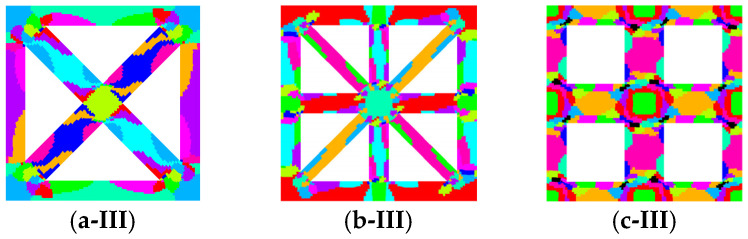
Microstructure of 2D lattice material. Configurations include the following: (**I**) 1 × 1 array, (**II**) 3 × 3 array, and (**III**) 30-cluster division, where each color represents a cluster; (**a**) x=0.00; (**b**) x=0.10; (**c**) x=0.20.

In this section, three different configurations from Section 3.1, specifically x=0.00, x=0.10, and x=0.20, are selected for the analysis of the plane strain problem. During the offline phase, the required strain concentration tensors are obtained by applying eigenstrains to the structure. The k-means method is used to divide each configuration into 30 clusters. The strain loads are then applied to each cluster. For the plane strain problem, the applied strain load is $\varepsilon =\left[\varepsilon_{xx},\varepsilon_{yy},\varepsilon_{zz},\varepsilon_{xy}\right]$, and the extracted stress components for each element are $\sigma =\left[\sigma_{xx},\sigma_{yy},\sigma_{zz},\sigma_{xy}\right]$. Each cluster consists of four components resulting in a 2D lattice material interaction matrix with dimensions $D^{120\times 120}$. The specific structural forms and clustering results are shown in Figure 8.

A linear elasticity analysis was performed on a 2D complex lattice material using the obtained interaction matrix. The results of the effective elasticity matrix and the anisotropy map of the elastic modulus are presented in Table 1. A comparison reveals that the effective elastic matrices of the 2D lattice material calculated by FCA and NIAH are very close, thus verifying the correctness of the calculation (see Appendix A for the NIAH implementation method). In addition, as parameter *x* increases, the first value in the effective elasticity matrix gradually increases, whereas the last value decreases. In the anisotropy map of the elastic modulus, the maximum modulus values increase with *x* and are 71.15 MPa, 81.15 MPa, and 93.32 MPa, respectively. Furthermore, the overall anisotropy of the structure evolves from a uniform distribution to a more pronounced convex–concave pattern. When x=0.00, the effective elastic modulus was relatively balanced in different directions, with the maximum and minimum values differing by only 3.02%. However, when x=0.20, the variation in the modulus across the different axes was significant, with the difference between the diagonal and coordinate axes reaching 61.65%. Therefore, the effective elastic modulus extremes and their distributions across the axes differ for different lattice materials. Lattice materials can be selected according to the requirements for the effective modulus and anisotropy.

Before conducting an online phase analysis of the above microstructures, the interaction matrix **D** must be reconstructed. The reconstruction strategy is based on Equations (17) and (18), which calculate the effective stiffness variation after removing the characteristic values. Based on the calculation method from a previous study [41], for x=0.00, 0.10, and 0.20, the truncation parameter is selected as 40, 40, and 42, respectively.

**Figure 9:**
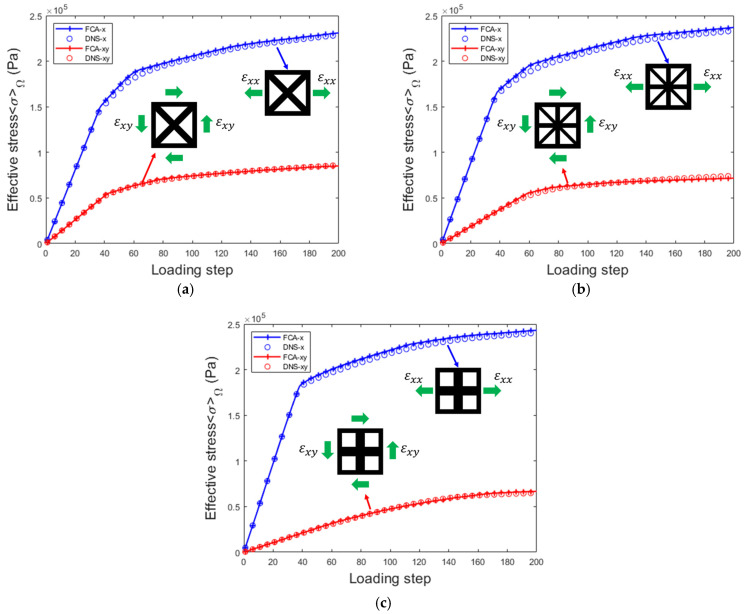
Effective stress–strain of FCA and DNS in 2D lattice materials under tensile and shear loads: (**a**) x=0.00; (**b**) x=0.10; (**c**) x=0.20.

In the online computation phase, two load cases are considered: uniaxial tension along the x-direction, represented by $\varepsilon_{1}=\left[0.01,0,0,0\right]$, and shear in the xy-plane, represented by $\varepsilon_{2}=\left[0,0,0,0.01\right]$. The number of load steps is set to 200. Figure 9 shows the comparison of the effective stress results obtained from the FCA and DNS methods for each load step. For each structure, the magnitude and trend of the results remained consistent, demonstrating the accuracy and applicability of the FCA method.

**Figure 10:**
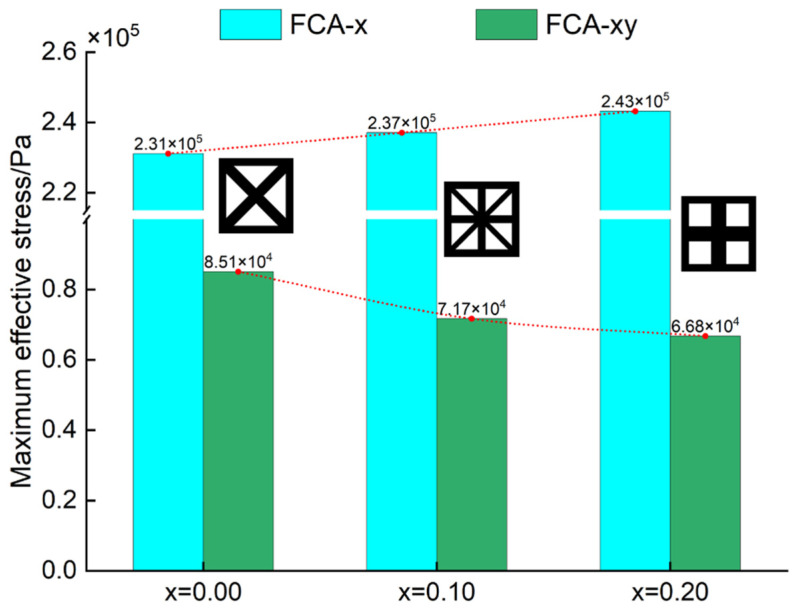
RVE maximum effective stress calculated by FCA.

Furthermore, the maximum effective stress values under different loading conditions for the three structural configurations were extracted and plotted in Figure 10. The comparison reveals that the maximum effective stress under tensile loading gradually increases with the structural parameter *x*, whereas the maximum effective stress under shear loading decreases. This trend is closely related to the diagonal support of the structure. Therefore, in structural load-bearing applications, when the shear stress is significant, lattice materials corresponding to x=0.00 should be selected, whereas for higher tensile stress, the structure corresponding to x=0.20 should be chosen, thus enabling better structural design. The FCA method can be used to quickly evaluate this nonlinear effective property.

Finally, an example is presented using a structure with parameter x=0.00. In this case, the computational time for the online calculations is compared with DNS. Specifically, the online FCA algorithm (excluding offline clustering and interaction matrix construction) is compared with DNS (excluding text reading/writing, which consumes significant computational time) over 200 strain-loading steps. The comparison focuses on the computation time for each loading step. The results shown in Table 2 reveal that the FCA method is 393 times more efficient than DNS for online calculations. This demonstrates the ability to quickly predict the nonlinear effective properties of two-dimensional lattice materials, confirming the feasibility of rapid nonlinear analysis for lattice materials.

### 4.2. 3D Results of Effective Properties Analysis

**Figure 11:**
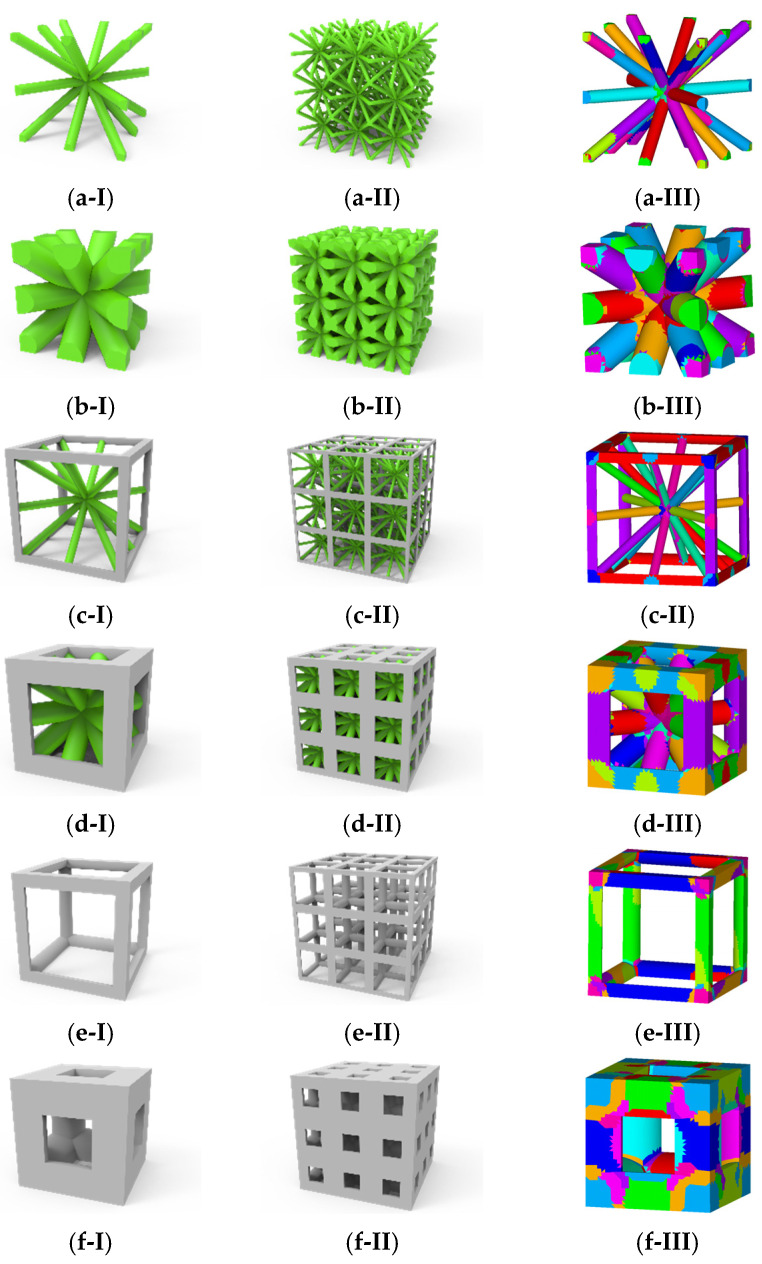
Microstructure of 3D lattice materials. Structures are arranged in patterns: (**I**) 1 × 1 array; (**II**) 3 × 3 array; (**III**) 30-cluster partitioning, where each color represents a cluster; (**a**) β=0.1, $\zeta_{exter}=0$; (**b**) β=0.5, $\zeta_{exter}=0$; (**c**) β=0.1, $\zeta_{exter}=0.5$; (**d**) β=0.5, $\zeta_{exter}=0.5$; (**e**) β=0.1, $\zeta_{exter}=1$; (**f**) β=0.5, $\zeta_{exter}=1$.

Based on the characteristics of the parametric modeling, this section considers six different 3D complex lattice material models: (1) β=0.1, $\zeta_{exter}=0$; (2) β=0.5, $\zeta_{exter}=0$; (3) β=0.1, $\zeta_{exter}=0.5$; (4) β=0.5, $\zeta_{exter}=0.5$; (5) β=0.1, $\zeta_{exter}=1$; and (6) β=0.5, $\zeta_{exter}=1$. For each lattice material, a clustering operation is performed for 30 clusters with six stress components extracted from each element. Therefore, the size of the interaction matrix is $D^{180\times 180}$. The specific lattice materials and clustering results for the six models are shown in Figure 11.

Using Equation (18), the interaction matrix is processed to obtain an effective elastic matrix for the 3D lattice material. The comparison shows that there is almost no difference in the equivalent elasticity matrix calculated by FCA and NIAH, which confirms the correctness of the calculation (see Appendix A for the NIAH implementation method). The lattice materials considered in this study exhibited geometric symmetry, and the components of their effective elastic matrices satisfy the following relationships: $C_{11}=C_{22}=C_{33}$, $C_{12}=C_{13}=C_{23}$, and $C_{44}=C_{55}=C_{66}$. The components $C_{11}$, $C_{12}$, and $C_{44}$ can be used to describe structures with three independent elastic constants. The isotropy of the structure increases as the shape of the calculated elastic modulus anisotropy diagram becomes more spherical. In Table 3, increasing the aspect ratio at constant relative density results in little overall change in the three independent elastic constants, although their individual change trends are different. Specifically, $C_{11}$ gradually increases, whereas $C_{12}$ and $C_{44}$ decrease, leading to significant changes in the anisotropic distribution. Increasing the relative density at a constant aspect ratio causes all three independent elastic constants to increase significantly, thereby reducing the degree of anisotropy.

Previous studies [51,52] introduced an anisotropy index, A, to describe the anisotropy of cubic crystal systems. The anisotropy index for the three axial directions is calculated as follows:

(32) $$A_{x}=\frac{2C_{44}}{C_{11}-C_{12}},\;\;\;\;\;\;\;\;\;\;\;A_{y}=\frac{2C_{55}}{C_{22}-C_{23}},\;\;\;\;\;\;\;\;\;\;\;A_{z}=\frac{2C_{66}}{C_{33}-C_{13}}$$

where $A_{x}$, $A_{y}$, and $A_{z}$ are the anisotropy indices of the lattice material in the local coordinate directions x, y, and z, respectively. The cubic crystals become more isotropic as these values get closer to the value 1.

**Figure 12:**
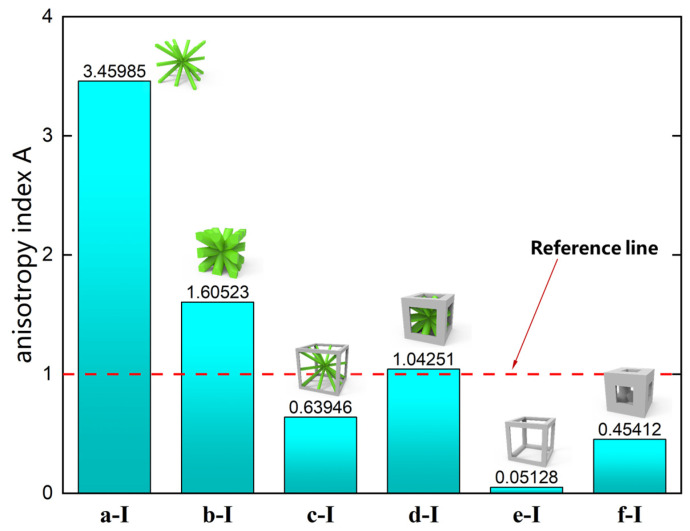
Anisotropy index of six complex microstructure lattice materials.

It is evident that the six structures are symmetric models, meaning $A_{x}$ = $A_{y}$ = $A_{z}$. Using the above formula, the anisotropy indices for these six structures were calculated, and the results shown in Figure 12 are like the patterns observed in the elastic modulus anisotropy charts.

**Figure 13:**
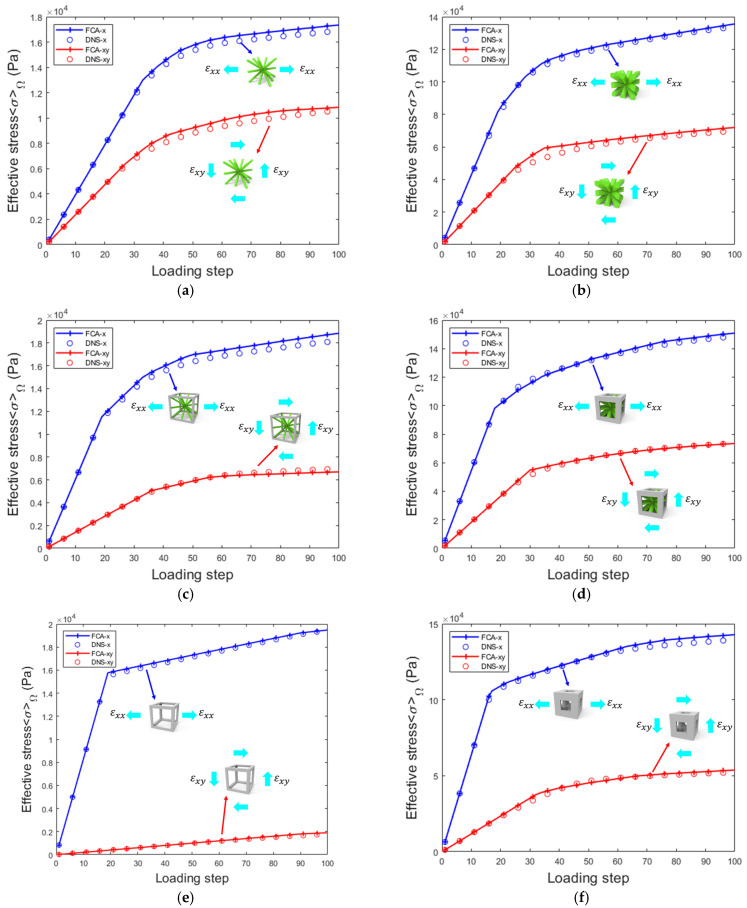
Effective stress–strain of FCA and DNS in 3D lattice materials under tensile and shear loads: (**a**) β=0.1 and $\zeta_{exter}=0$; (**b**) β=0.5 and $\zeta_{exter}=0$; (**c**) β=0.1 and $\zeta_{exter}=0.5$; (**d**) β=0.5 and $\zeta_{exter}=0.5$; (**e**) β=0.1 and $\zeta_{exter}=1$; (**f**) β=0.5 and $\zeta_{exter}=1$.

In the online analysis phase, 100 load steps were applied, with the x-direction tensile loading conditions $\varepsilon_{1}=\left[0.01,0,0,0,0,0\right]$ and xy shear loading conditions $\varepsilon_{2}=\left[0,0,0,0,0,0.01\right]$. As shown in Figure 13, a comparison of the results from the FCA and DNS shows that each structure exhibits consistent trends with minimal numerical differences. The FCA method provided a good fit for solving the nonlinear effective stress of the corresponding RVE, demonstrating its applicability in computing 3D lattice materials.

**Figure 14:**
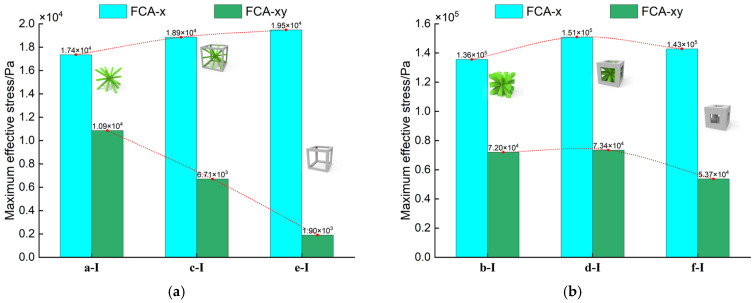
Maximum effective stress of 3D lattice materials calculated by FCA under tensile and shear loads at different relative densities: (**a**) β=0.1; (**b**) β=0.5.

Next, the changes in the maximum stress of the structures with different aspect ratios at constant relative density are compared. As shown in Figure 14, when the relative density β=0.1, the maximum effective tensile stress increases with the aspect ratio, while the maximum effective shear stress decreases. This trend allows the selection of lattice materials based on the requirements of tensile and shear stresses. However, when the relative density β=0.5, both the maximum effective tensile stress and shear stress first increase and then decrease as the aspect ratio increases. This anomaly can be explained by the fact that, at higher relative densities, the material distribution within the structure becomes more uniform. The coordination of the central diagonal struts and surrounding rods allows for better overall structural performance.

Finally, a lattice material with parameters β=0.1 and $\zeta_{exter}=0$ is analyzed to evaluate its online computational time. The time required for the FCA online algorithm (excluding offline clustering and interaction matrix construction) and DNS (excluding text reading and writing, which consume substantial computation time) is evaluated over 100 strain-loading steps. The results in Table 4 show that the FCA method improves efficiency by a factor of 11,702 compared with DNS. This demonstrates the effectiveness of the FCA approach for quickly predicting the nonlinear effective properties of 3D lattice materials, confirming the feasibility of the rapid nonlinear analysis of lattice materials.

### 4.3. Comparative Analysis Using Voxel Elements

**Figure 15:**
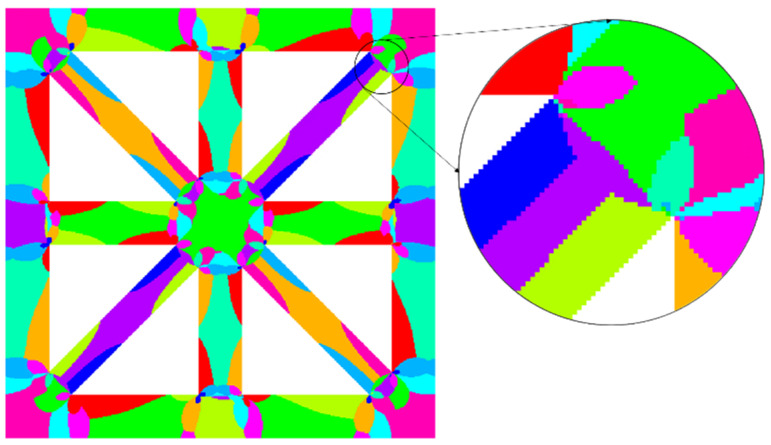
Schematic diagram of voxel-based lattice material clustering and partitioning, where each color represents a cluster.

In Section 3.3, offline calculations were performed on the VG models. The k-means clustering algorithm was used to divide the structure into 30 clusters, the specific details of which are shown in Figure 15.

**Figure 16:**
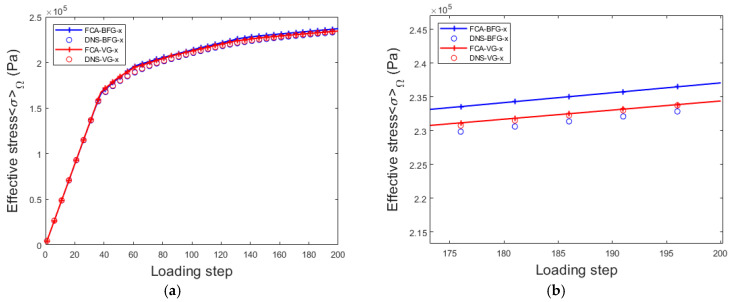
Effective stress for voxel grid (VG) and body-fitted grid (BFG) models for lattice materials: (**a**) steps 0–200; (**b**) steps 175–200.

Based on the offline database established through clustering analysis, the online FCA algorithm was used to perform a nonlinear analysis of the voxel-modeled lattice material. A load of ε=[0.01,0,0,0] was applied, and the results were compared with those obtained from DNS calculations. By combining the analysis in Section 4.1, the differences in the curves between the VG and body-fitted grid (BFG) modeling methods are examined. A comparison of the curves is shown in Figure 16.

The comparison of the results reveals the following insights. First, the values obtained from the VG and BFG modeling are similar. According to the modeling parameters in Section 3.3, the volume fraction ratio of the voxel modeling is 0.6424 and that of the non-voxel modeling was 0.64. The difference between the two is small, indicating that the effective properties of the two lattices are similar. Second, during the nonlinear analysis phase of voxel modeling, the details show that the FCA results closely matched the DNS results. The regular grid structure in voxel modeling allows for a more accurate and effective property estimation during FCA online analysis.

Finally, when comparing the online computation times, although the VG model had 327,954 degrees of freedom and the BFG model had 30,418 degrees of freedom when both are clustered into 30 blocks, the computational efficiency of the two modeling methods is similar, with a time consumption of 0.588 s. The next step in this study will be to investigate how to combine voxel modeling with the fast interaction matrix algorithm from a previous study [50]. These findings provide valuable support for future research in this field.

## 5. Conclusions and Outlooks

In this study, the FCA approach is used, focusing on lattice materials. The FCA method is extended to the rapid elastoplastic effective properties of complex micro structured lattice materials, leveraging its advantages in porous material calculations. Based on the above analysis, the following conclusions were drawn.

(1) Unlike prior investigations on fiber- and particle-reinforced composites, complex micro structured lattice materials were constructed using parametric modeling methods. FCA was used to rapidly determine the differences in the effective stiffness and stress–strain curves of the lattice materials for different parameter values. By integrating the effective stiffness matrix, elastic modulus anisotropy diagrams are generated to clarify the effective stiffness and microstructural anisotropy characteristics of lattice materials from a micromechanical perspective;
(2) Through extensive RVE examples of complex microstructure lattice materials, the effectiveness of the FCA in predicting complex microstructure lattice materials was validated by combining offline and online algorithms. The accuracy and efficiency of the FCA were compared with those of the DNS, thereby further highlighting its advantages;
(3) In this study, the differences in FCA prediction outcomes between voxel and non-voxel models were investigated. For lattice materials with the same microstructure and porosity, online FCA calculations based on voxel modeling can achieve more accurate and effective attribute estimation.

These results indicate that the FCA method can quickly and effectively predict the nonlinear effective properties of lattice materials. Based on this foundation, in the future, this computational advantage will be combined to perform a two-scale elastoplastic nonlinear analysis of lattice materials, elastoplastic topology optimization design considering lattice materials, and relevant experimental techniques for lattice material analysis [53,54], thus providing a solid foundation for better use of lattice materials in structural design.

## Figures and Tables

**Table 1:** Statistical table of effective elasticity matrix and corresponding elastic modulus anisotropy map for 2D lattice microstructure. (Note: the NIAH method calculates material properties based on node force and displacement, which precludes derivation of z-direction values for plane strain elements; omitted results are indicated by “−”).

Parameter	Microstructure	Effective Elastic Matrix C (MPa)		Elastic Modulus Anisotropy Diagram (MPa)
x=0.00	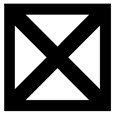	FCA	$\left[\begin{array}{cccc} 80.85 & 30.95 & 33.54 & 0\\ 30.95 & 80.85 & 33.54 & 0\\ 33.54 & 33.54 & 148.13 & 0\\ 0 & 0 & 0 & 26.09 \end{array}\right]$	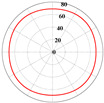
		NIAH	$\left[\begin{array}{cccc} 80.85 & 30.95 & - & 0\\ 30.95 & 80.85 & - & 0\\ - & - & - & -\\ 0 & 0 & - & 26.09 \end{array}\right]$	−
x=0.10	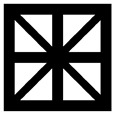	FCA	$\left[\begin{array}{cccc} 88.35 & 25.22 & 34.07 & 0\\ 25.22 & 88.35 & 34.07 & 0\\ 34.07 & 34.07 & 148.44 & 0\\ 0 & 0 & 0 & 18.74 \end{array}\right]$	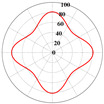
		NIAH	$\left[\begin{array}{cccc} 88.31 & 25.20 & - & 0\\ 25.20 & 88.31 & - & 0\\ - & - & - & -\\ 0 & 0 & - & 18.74 \end{array}\right]$	−
x=0.20	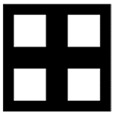	FCA	$\left[\begin{array}{cccc} 97.55 & 20.31 & 35.36 & 0\\ 20.31 & 97.55 & 35.36 & 0\\ 35.36 & 35.36 & 149.21 & 0\\ 0 & 0 & 0 & 10.55 \end{array}\right]$	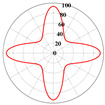
		NIAH	$\left[\begin{array}{cccc} 97.54 & 20.31 & - & 0\\ 20.31 & 97.54 & - & 0\\ - & - & - & -\\ 0 & 0 & - & 10.55 \end{array}\right]$	−

**Table 2:** Table of computational time for 2D lattice microstructure with parameter x=0.00.

	FCA	DNS
Number of degrees of freedom	120	29,282
Computational time (one step)	0.003 s	1.179 s
Contrast	393 times	-

**Table 3:** Statistical table for effective elasticity matrix and corresponding elasticity modulus anisotropy of lattice microstructure.

Parameter	Microstructure	Effective Elastic Matrix C (MPa)		Elastic Modulus Anisotropy Diagram
β=0.1 $\zeta_{exter}=0$	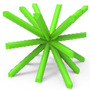	FCA	$\left[\begin{array}{cccccc} 3.94 & 2.57 & 2.57 & 0 & 0 & 0\\ 2.57 & 3.94 & 2.57 & 0 & 0 & 0\\ 2.57 & 2.57 & 3.94 & 0 & 0 & 0\\ 0 & 0 & 0 & 2.37 & 0 & 0\\ 0 & 0 & 0 & 0 & 2.37 & 0\\ 0 & 0 & 0 & 0 & 0 & 2.37 \end{array}\right]$	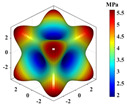
		NIAH	$\left[\begin{array}{cccccc} 3.94 & 2.56 & 2.56 & 0 & 0 & 0\\ 2.56 & 3.94 & 2.56 & 0 & 0 & 0\\ 2.56 & 2.56 & 3.94 & 0 & 0 & 0\\ 0 & 0 & 0 & 2.37 & 0 & 0\\ 0 & 0 & 0 & 0 & 2.37 & 0\\ 0 & 0 & 0 & 0 & 0 & 2.37 \end{array}\right]$	−
β=0.5 $\zeta_{exter}=0$	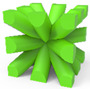	FCA	$\left[\begin{array}{cccccc} 42.84 & 19.13 & 19.13 & 0 & 0 & 0\\ 19.13 & 42.84 & 19.13 & 0 & 0 & 0\\ 19.13 & 19.13 & 42.84 & 0 & 0 & 0\\ 0 & 0 & 0 & 19.03 & 0 & 0\\ 0 & 0 & 0 & 0 & 19.03 & 0\\ 0 & 0 & 0 & 0 & 0 & 19.03 \end{array}\right]$	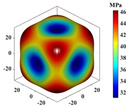
		NIAH	$\left[\begin{array}{cccccc} 42.66 & 19.10 & 19.10 & 0 & 0 & 0\\ 19.10 & 42.66 & 19.10 & 0 & 0 & 0\\ 19.10 & 19.10 & 42.66 & 0 & 0 & 0\\ 0 & 0 & 0 & 19.03 & 0 & 0\\ 0 & 0 & 0 & 0 & 19.03 & 0\\ 0 & 0 & 0 & 0 & 0 & 19.03 \end{array}\right]$	−
β=0.1 $\zeta_{exter}=0.5$	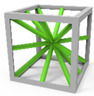	FCA	$\left[\begin{array}{cccccc} 6.06 & 1.65 & 1.65 & 0 & 0 & 0\\ 1.65 & 6.06 & 1.65 & 0 & 0 & 0\\ 1.65 & 1.65 & 6.06 & 0 & 0 & 0\\ 0 & 0 & 0 & 1.41 & 0 & 0\\ 0 & 0 & 0 & 0 & 1.41 & 0\\ 0 & 0 & 0 & 0 & 0 & 1.41 \end{array}\right]$	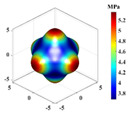
		NIAH	$\left[\begin{array}{cccccc} 6.06 & 1.65 & 1.65 & 0 & 0 & 0\\ 1.65 & 6.06 & 1.65 & 0 & 0 & 0\\ 1.65 & 1.65 & 6.06 & 0 & 0 & 0\\ 0 & 0 & 0 & 1.41 & 0 & 0\\ 0 & 0 & 0 & 0 & 1.41 & 0\\ 0 & 0 & 0 & 0 & 0 & 1.41 \end{array}\right]$	−
β=0.5 $\zeta_{exter}=0.5$	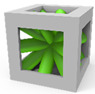	FCA	$\left[\begin{array}{cccccc} 54.56 & 19.51 & 19.51 & 0 & 0 & 0\\ 19.51 & 54.56 & 19.51 & 0 & 0 & 0\\ 19.51 & 19.51 & 54.56 & 0 & 0 & 0\\ 0 & 0 & 0 & 18.27 & 0 & 0\\ 0 & 0 & 0 & 0 & 18.27 & 0\\ 0 & 0 & 0 & 0 & 0 & 18.27 \end{array}\right]$	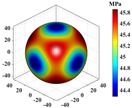
		NIAH	$\left[\begin{array}{cccccc} 54.56 & 19.50 & 19.50 & 0 & 0 & 0\\ 19.50 & 54.56 & 19.50 & 0 & 0 & 0\\ 19.50 & 19.50 & 54.56 & 0 & 0 & 0\\ 0 & 0 & 0 & 18.27 & 0 & 0\\ 0 & 0 & 0 & 0 & 18.27 & 0\\ 0 & 0 & 0 & 0 & 0 & 18.27 \end{array}\right]$	−
β=0.1 $\zeta_{exter}=1$	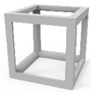	FCA	$\left[\begin{array}{cccccc} 8.30 & 0.50 & 0.50 & 0 & 0 & 0\\ 0.50 & 8.30 & 0.50 & 0 & 0 & 0\\ 0.50 & 0.50 & 8.30 & 0 & 0 & 0\\ 0 & 0 & 0 & 0.2 & 0 & 0\\ 0 & 0 & 0 & 0 & 0.2 & 0\\ 0 & 0 & 0 & 0 & 0 & 0.2 \end{array}\right]$	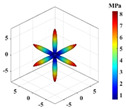
		NIAH	$\left[\begin{array}{cccccc} 8.30 & 0.50 & 0.50 & 0 & 0 & 0\\ 0.50 & 8.30 & 0.50 & 0 & 0 & 0\\ 0.50 & 0.50 & 8.30 & 0 & 0 & 0\\ 0 & 0 & 0 & 0.2 & 0 & 0\\ 0 & 0 & 0 & 0 & 0.2 & 0\\ 0 & 0 & 0 & 0 & 0 & 0.2 \end{array}\right]$	−
β=0.5 $\zeta_{exter}=1$	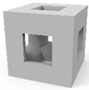	FCA	$\left[\begin{array}{cccccc} 63.82 & 12.16 & 12.16 & 0 & 0 & 0\\ 12.16 & 63.82 & 12.16 & 0 & 0 & 0\\ 12.16 & 12.16 & 63.82 & 0 & 0 & 0\\ 0 & 0 & 0 & 11.73 & 0 & 0\\ 0 & 0 & 0 & 0 & 11.73 & 0\\ 0 & 0 & 0 & 0 & 0 & 11.73 \end{array}\right]$	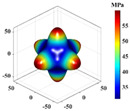
		NIAH	$\left[\begin{array}{cccccc} 63.82 & 12.16 & 12.16 & 0 & 0 & 0\\ 12.16 & 63.82 & 12.16 & 0 & 0 & 0\\ 12.16 & 12.16 & 63.82 & 0 & 0 & 0\\ 0 & 0 & 0 & 11.73 & 0 & 0\\ 0 & 0 & 0 & 0 & 11.73 & 0\\ 0 & 0 & 0 & 0 & 0 & 11.73 \end{array}\right]$	−

**Table 4:** Computation time statistics for 3D lattice microstructure with β=0.1 and $\zeta_{exter}=0$.

	FCA	DNS
Number of degrees of freedom	180	332,787
Computational time	0.277 s	3241.7 s
Contrast	11,702 times	-

**Figure A1:**
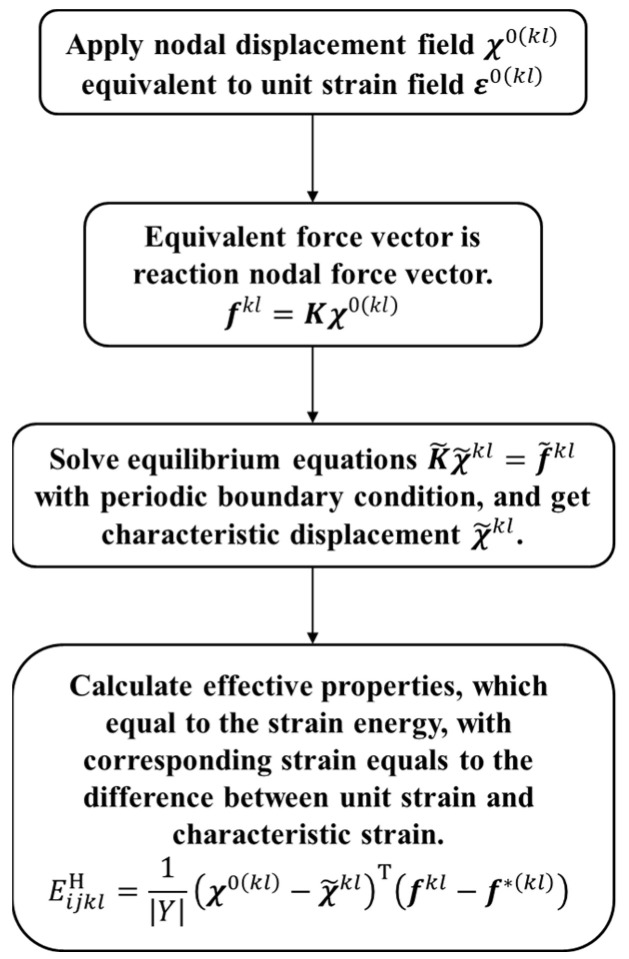
Flowchart for calculating effective properties using NIAH method.

## Data Availability

The original contributions presented in this study are included in the article. Further inquiries can be directed to the corresponding authors.

## Abbreviations

The following acronyms are used in this manuscript:

FCA	FEM-Cluster-based Analysis
DNS	Direct Numerical Simulation
RVE	Representative Volume Element
NIAH	Novel Implementation of Asymptotic Homogenization
FEM	Finite Element Method
FFT	Fast Fourier Transform
POD	Proper Orthogonal Decomposition
PGD	Proper Generalized Decomposition
TFA	Transformation Field Analysis
NTFA	Nonuniform Transformation Field Analysis
SCA	Self-consistent Clustering Analysis
VCA	Virtual Clustering Analysis
DNN	Deep Neural Network
LSTM	Long Short-Term Memory
PCMCE	Principle of Cluster Minimum Complementary Energy
2D	Two-dimensional
3D	Three-dimensional
FEA	Finite Element Analysis
VG	Voxel Grid
BFG	Body-fitted Grid

## Nomenclature

Explanation of all quantities used in this paper.

σ	Stress
ε	Strain
$\Omega_{1}$	The matrix domain
$\Omega_{2}$	The pore domain
Ω	The entire domain
u	Displacement
$\partial \Omega ,\partial \Omega_{1},\partial \Omega_{2}$	The corresponding boundary differential part
$\Delta \Phi^{n}$	The increment of porous strain energy and effective stress
C(x)	The elastic stiffness matrix
$f\left(x,\varepsilon^{\prime },\omega \right)$	Nonlinear function
ω	The parameters corresponding to various plastic strengthening criteria
$\varepsilon^{\prime }$	The mechanical strain
$\varepsilon^{*}$	Eigenstrain
A	The strain concentration tensor
K	The total stiffness matrix of the RVE
$U^{mn}$	The nodes displacement vector
$F^{mn}$	The “external force” vector applied at the nodes of the RVE
$\varepsilon^{\mathrm{micro}}\left(x\right)$	The elastic microscopic strain
$\varepsilon^{\mathrm{macro}}\left(X\right)$	The homogeneously elastic macroscopic strain
Y	Minimizing the total distance
$A_{e}$	The strain concentration tensor for the e-th element
${\bar{A}}^{I}$	The average strain concentration tensor for all elements in the I-th cluster $\Omega^{I}$
$n_{c}$	The total number of clusters
D	Interaction matrix
$D_{ijkl}^{IJ}$	Interaction matrix components
$\sigma_{ij,e}^{\left(\Psi \right)}$	The stress vectors of each cluster in the cluster model
${\hat{\varepsilon }}_{\Lambda ,kl}^{*\left(\Psi \right),J}$	The eigenstrain of the unit cluster under the applied working condition Ψ
$\Lambda^{D}$	The singular value matrix
Q,P	The orthogonal matrices of the singular vectors of matrix D
$D^{*}$	Reconstructed interaction matrix
α	Column vector
$S^{\prime }$	Elastoplastic compliance matrix
V	The volume diagonal matrix of each cluster
$\Delta {\hat{\sigma }}_{k}$	The stress increment of step k
${\hat{\sigma }}_{k}$	The stress of step k
${\hat{\sigma }}_{k+1}$	The stress increment of step *k* + 1
β	The relative density
$V_{strut}$	The volume of the internal struts that form the lattice microstructure
$V_{lattice}$	The envelope volume enclosed by the external boundary of the lattice microstructure
r	The radius of the framework
d	The diameter of the struts
$\zeta_{exter}$	The aspect ratios
$V_{exter}$	The volume of the external frame
A	Anisotropy index

## Appendix A

Referring to references [19,20], the calculation process for NIAH is as follows:
